# CDCS: Cluster-Based Distributed Compressed Sensing to Facilitate QoS Routing in Cognitive Video Sensor Networks

**DOI:** 10.3390/e21040345

**Published:** 2019-03-28

**Authors:** Hang Shen, Lingli Li, Tianjing Wang, Guangwei Bai

**Affiliations:** 1 College of Computer Science and Technology, Nanjing Tech University, Nanjing 211816, China; 2 National Engineering Research Center of Communications and Networking, Nanjing University of Posts and Telecommunications, Nanjing 210003, China

**Keywords:** spatial correlation, quality-of-service, distributed compressed sensing, information theory, cognitive video sensor networks

## Abstract

Compressed sensing based in-network compression methods which minimize data redundancy are critical to cognitive video sensor networks. However, most existing methods require a large number of sensors for each measurement, resulting in significant performance degradation in energy efficiency and quality-of-service satisfaction. In this paper, a cluster-based distributed compressed sensing scheme working together with a quality-of-service aware routing framework is proposed to deliver visual information in cognitive video sensor networks efficiently. First, the correlation among adjacent video sensors determines the member nodes that participate in a cluster. On this basis, a sequential compressed sensing approach is applied to determine whether enough measurements are obtained to limit the reconstruction error between decoded signals and original signals under a specified reconstruction threshold. The goal is to maximize the removal of unnecessary traffic without sacrificing video quality. Lastly, the compressed data is transmitted via a distributed spectrum-aware quality-of-service routing scheme, with an objective of minimizing energy consumption subject to delay and reliability constraints. Simulation results demonstrate that the proposed approach can achieve energy-efficient data delivery and reconstruction accuracy of visual information compared with existing quality-of-service routing schemes.

## 1. Introduction

Wireless video sensor networks (WVSNs) [1] refer to networks of interconnected video sensor nodes equipped with audio and video information collection modules. Such networks can process, transmit and fuse multimedia data originated from heterogeneous sources. Quality-of-Service (QoS) sensitive video applications require more spectrum and transmission resources [2,3,4,5,6,7]. Cognitive radio (CR) [8] as a promising technology has been presented to improve spectrum utilization. Some CR standards have been developed in TV White Spaces (TVWS) band [9]. Secondary users (SUs) can opportunistically access the TVWS while preventing harmful interference to primary user (PU) transmissions. CR can also be applied to WVSNs for better communication performance, which evolves into cognitive video sensor networks (CVSNs) [10,11] aimed at delivering video content with a predetermined level of QoS. However, in addition to resource constraints such as limited energy and processing capacity, the transmission of visual information requires high-bandwidth, high-fidelity and more processing energy. These challenges and constraints, along with the complex network environment, make video transmission and processing over CVSNs a challenging issue.

Typical applications in CVSNs require spatially dense sensor deployment to achieve satisfactory coverage [12]. As a consequence, multiple sensors often record information about one event in each field-of-view (FoV). Due to the high density in the network topology, spatially proximal sensor observations are highly correlated, leading to much redundant visual data. Collaborative in-network multimedia processing [13] is promising for alleviating redundant data, with which each sensor can filter uninterested information or integrate observations with other correlated nodes. Unlike spatial correlation of user experience [14] which affects the performance of cooperative spectrum sensing, here we focus on the spatial correlation of visual information that exists among the observations of distributed video sensors with overlapped FoVs. By performing correlation-aware in-network video processing, the communication cost/bandwidth usage of the entire downstream path decreases at the expense of relatively small energy consumption induced by the local transmission and compression. This method facilitates a routing protocol to provide QoS guarantees.

Compressed sensing (CS) [15] as an extension of information theory can be introduced that deals with visual information for CVSNs. Such a technique is an alternative to the traditional Shannon–Nyquist sampling for sparse/compressible signal acquisition, aiming to extract as much information as possible from as little data as possible. Specifically, sampling devices collect not the original signal, but the data after orthogonal transformation. Each observation contains original signals to some extent and each part of an original signal diffuse in each observation. Distributed compressed sensing (DCS) [16,17,18] extends CS to exploit both intra- and inter-signal correlation for video compression, thereby reducing the amount of data for signal reconstruction. According to the theory of DCS, a signal can be reconstructed by a small number of linear observations as long as the signal can be represented sparsely on some bases. Many existing analyses characterize how many measurements we need for the signal with a given sparsity level. As the sparsity level is often not known a priori, obtaining the signal sparsity in practical applications is very difficult.

The focus of this work is on the energy-efficient transmission of visual information in the presence of QoS constraints in CVSNs. We propose a cluster-based distributed compressed sensing scheme (called CDCS) working together with a distributed QoS routing to facilitate delivery of video data. The main contributions are as follows:

- A correlation metric for adjacent video sensors with overlapped FoVs is utilized to determine which video sensor can participate in a cluster. The purpose is to enhance video compression efficiency and reduce transmission cost to the sink.
- A sequential compressed sensing (SCS) approach is explored to decide whether enough measurements are obtained to limit video reconstruction error to a specified threshold. With the approach, we select collaborative nodes to carry out DCS in a cluster without any a priori assumptions regarding signal sparsity.
- A distributed spectrum-aware QoS routing framework is presented to transmit the compressed video data with consideration of the condition for video frame decoding. The goal to minimize energy consumption subject to delay and reliability constraints.
- The effectiveness and superiority of CDCS are validated through extensive simulations. The results show that CDCS could achieve energy efficient QoS communication while maintaining acceptable image quality.

The remainder of this paper is structured as follows. Section 2 introduces background and existing research about in-network processing and QoS routing. Section 3 motivates this work. We present the implementation details including clustering, node collaboration, and route selection in Section 4. Performance evaluation is given in Section 5, followed by the conclusions in Section 6.

## 2. Background and Related Works

In this section, we analyze current in-network video processing techniques in wireless networks and discuss the characteristics and problems of existing QoS routing protocols in sensor networks.

Most of the existing works provide QoS guarantees by properly distributing network traffic, without considering the removal of unnecessary multimedia loads. In-network compression [19] can effectively reduce the number of packets transmitted in the network and guarantee the accurate reconstruction of compressed data at the sink. The work in [20] takes advantage of high spatial correlation of observation retrieved from proximal video sensor to process in-network video through differential coding, which can reduce a lot of redundant data in routing. The problem of how to choose relay nodes with differential coding to reduce redundant multimedia data in a multi-sink environment is taken into account in [20]. A centralized clustering algorithm is proposed in [21], in which cluster heads use a hybrid CS mechanism to transfer data to the sink through a backbone tree. However, it ignores the situation that sparse random matrices can be used in each cluster to reduce the number of packets transmitted. Ref. [22] presents a cluster-based data aggregation method with sparse random measurements in a star topology-based sensor network. Unfortunately, the cluster structure of star topology may lead to an increase in intra-cluster energy consumption. CBCA [23] is a greedy clustering algorithm on top of a logical chain to minimize the average compression ratio of all clusters. This work provides a valuable reference for hierarchical compressive data gathering in sensor networks.

Most of the prior works on QoS routing protocols in wireless sensor networks are designed to support two performance metrics: delay and reliability [24,25,26,27]. MMSPEED [28] takes a cross-layer design approach to distinguish the communication flows with different delay and reliability demands and provide end-to-end QoS guarantees. The primary problem for MMSPEED is that it lacks control for redundant data, so there is an increase in communication congestion. In [29], SCEEM is proposed for CRSNs that jointly overcomes the formidable limitations of energy and spectrum without damaging multimedia quality. However, the process of data transmission may interfere with and even affect the primary users’ transmission. EARQ [30] can provide real-time, reliable and energy-aware data delivery in industrial sensor networks, where the path selection depends on the local information estimation value. QMOR [25] is a QoS-aware multi-sink opportunistic routing protocol working together with differential coding for multimedia sensor networks. The authors in [31] propose a QoS and energy-aware dynamic routing protocol for multimedia sensor networks. This scheme creates multiple paths within an inclination angle and a certain range around the direction to the destination. Although these two protocols can deliver multimedia information under QoS constraints, they do not support CR and are not directly tailored for CVSNs.

Despite the reduction of communication cost with in-network video processing, there are still many challenges in delivering data subject to QoS constraint. An effective way to improve network performance is to combine DCS with QoS routing. After studying relevant literature, we realize that there is no comprehensive study concerning the joint design of DSC and spectrum-aware QoS routing in CVSNs.

## 3. Motivation

This section motivates the work from three aspects: (1) impact of correlation on compression efficiency, (2) the reason why DCS can better control redundancy, and (3) the impact of cluster head selection on routing.

### 3.1. Impact of Correlation on Compression Efficiency

In CVSNs, due to different sensing directions, video sensors have a different range of FoVs, and their observations have a different correlation. Video sensors with large overlapped FoVs are likely to report the same event concurrently, and they are likely to have high compressed sensing gains.

**Figure 1:**
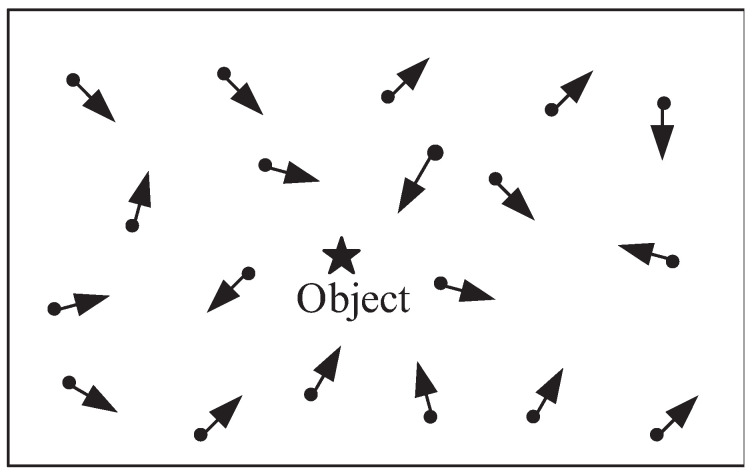
An interest object.

Take Figure 1 as an example to illustrate how the correlation affects compression efficiency. In CVSNs, the application specifies which object it is interested in, and the sensors that can observe this object may be members belonging to the same cluster. The overlapped ratio of FoVs determines in a substantial probability whether video sensors can observe the object of interest. There exists a high correlation among observations of the nodes with large overlapped FoVs. If video sensors with large overlapped FoVs belong to the same cluster, it is possible to achieve a good video compression effect. If we randomly cluster video sensor nodes without consideration of the correlation of FoVs, some nodes that do not observe the object may appear in the cluster. Because there are no targets observed, they fail to participate in compression, which makes application requirements hard to be satisfied.

### 3.2. Impact of Sparsity on Redundancy Removal

Due to the huge size of raw visual information, images and video sequences are compressed before transmission. We present an example to show the impact of DCS on the removal of redundancy. Consider a length-*N*, real-valued signal of one dimension indexed as *x*, its coefficient θ is sparse in wavelet basic ψ, i.e., x=ψθ, where θ is an N×1 column vector with *k* non-zero elements and *k* is the sparsity of θ.

**Figure 2:**
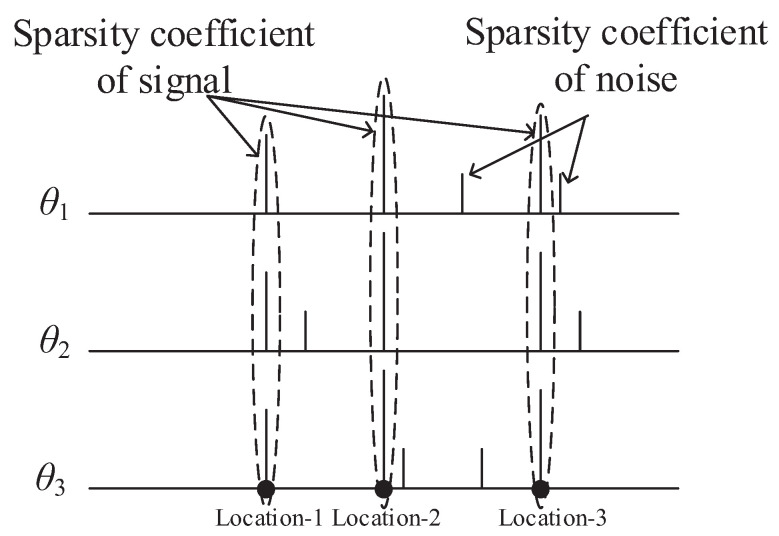
Sparsity coefficient of signals observed by adjacent sensors.

Take Figure 2 as an example of sparsity coefficients of signals observed by three different sensors, where the signals are processed under three situations, i.e., non-compression, CS and DCS. The horizontal direction (equivalent to *x*-axis) represents different locations of sparsity coefficients observed by different sensors; the vertical directions (equivalent to *y*-axis) represents different sparsity coefficients (i.e., $\theta_{1}$, $\theta_{1}$ and $\theta_{3}$). The locations of the sparsity coefficient are the same for different coefficients, but sparsity coefficients of noise are different. Consider a simple case where there is no encoder to process these three signals. If the length of a signal is 40, 120 signal samples need to be transmitted for the three signals. Next, consider a simple case where there is an encoder to process these three signals by CS. Based upon the CS machinery, M=c·k (c=4) measurements are required to reconstruct the signal *x*. Accordingly, we need to transmit 60 signal samples for three signals. If the encoder processes these three signals by DCS, $M=c\cdot (K+\sum_{i}k_{i})$ measurements are expected to reconstruct the signal *x*, where *K* represents the common sparsity of three coefficients and $k_{i}$ represents the unique sparsity of coefficient $\theta_{i}$. As a result, only 36 signal samples are transmitted for these three signals.

The above analysis indicates that DCS can mainly reduce unnecessary redundancy of data transmission compared with compressed sensing. However, if *m* sensors can realize the application requirement, there is no need to let all sensors that observe the object of interest compress signal. By applying SCS [32], *m* sensors can be selected out of *n* sensors to take part in DCS, which can make the reconstruction error below the defined threshold and further reduce the communication load.

### 3.3. Impact of Cluster Head Selection on QoS Routing

A cluster head acts as the source node to send compressed data. Not only the distance between a cluster head and the place where the event occurs but also the potential energy consumption should be considered in cluster head selection. Due to the existence of PUs, the channel availability of each node is different. If a cluster head is located in PUs’ transmission range and the PUs are occupying its data channel, its data transmission process is postponed, and many packets may be stacked on a cluster head waiting to be sent. Once the channel is detected to be released by the PUs, the cluster head may generate a large amount of traffic in a short time. These backlogs exacerbate network congestion and make it difficult for a routing protocol to meet QoS requirements or to consume more energy to achieve this goal.

## 4. Cluster-Based Distributed Compressed Sensing for QoS Routing

**Figure 3:**
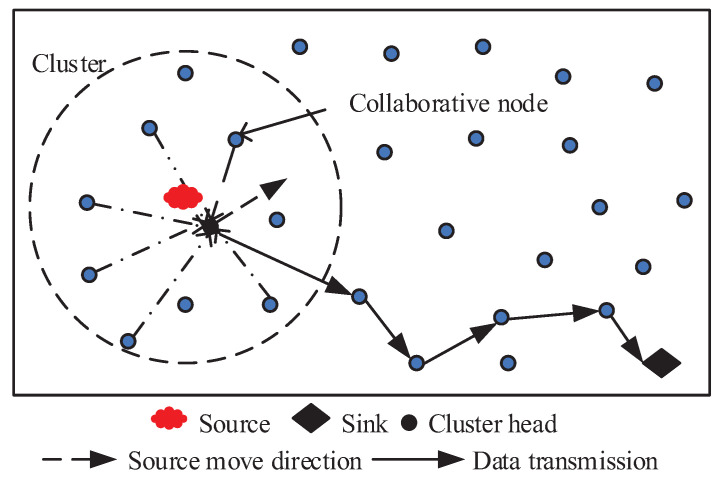
Cluster-based distributed compressed sensing framework for QoS routing.

This section presents a cluster-based distributed compressed sensing approach for QoS routing, the primary mechanism of which is comprised of the following parts (shown in Figure 3):

1. Event-driven clustering: A video sensor is triggered, and the clustering process is generated when an event is detected within their vicinity. The cluster consisting of several member nodes is formed in the dashed circles.
2. Collaborative node selection based on SCS: After a cluster is formed, the sink sends a message to the cluster head to inform the reconstruction error. On this basis, the cluster head uses the SCS approach to determine how many collaborative nodes are selected to participate in DCS to meet the requirement of reconstruction error rate.
3. QoS-aware routing selection: Each node respectively selects the optimal next hop with the objective of minimizing energy consumption and satisfying QoS requirements in delay and reliability. Afterward, compressed data is transmitted to the sink along the chosen path.

### 4.1. Event-Driven Clustering

#### 4.1.1. Problem Formulation

**Figure 4:**
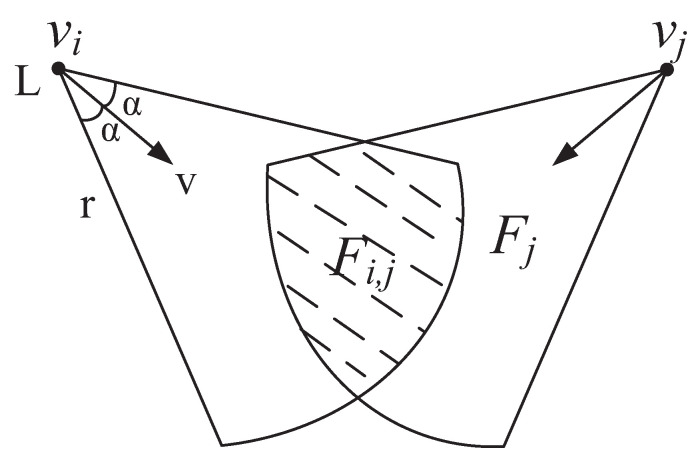
Overlapped FoVs.

Consider a CVSN containing *N* homogeneous video sensor nodes denoted by $S=\{v_{i}|i=1,2,\ldots ,N\}$ and some ordinary nodes used for detecting events. Both video sensors and scalar nodes are cognitive radio nodes; they and primary users coexist in CVSN. Video sensors can only observe the object in their FoVs. As shown in Figure 4, the FoVs of video sensor is determined by four parameters: the location of the video sensor (*L*), the sensing radius (*r*), the sensing direction (*v*) and the offset angle (α). It is assumed that sensing direction *v* of all nodes is fixed. Suppose that there are two sensors $v_{i}$ and $v_{j}$ with FoVs $F_{i}$ and $F_{j}$. Let $X_{i}$ and $X_{j}$ denote their observed images, both of which are correlated if $F_{i}$ and $F_{j}$ are overlapped with each other.

Two metrics which characterize the correlation between adjacent video sensors are introduced to select member nodes.

The overlapped ratio of FoVs for $v_{i}$ and $v_{j}$, denoted by $r_{i,j}$, is defined as

(1) $$r_{i,j}=\frac{S\left(F_{i,j}\right)}{S\left(F_{i}\right)},$$

where $S\left(F_{i,j}\right)\left(F_{i,j}=F_{i}\cap F_{j}\right)$ is the overlapped area of $F_{i}$ and $F_{j}$, and $S\left(F_{i}\right)$ is the area of $F_{i}$. If two video sensors have large overlapped ratio of FoVs, large portions of the two observed images are correlated, and they are likely to observe the same event concurrently.

Consider two adjacent nodes $v_{i}$ and $v_{j}$ with FoVs $F_{i}$ and $F_{j}$ that can observe an object of interest. Suppose both nodes capture the image about the object of interest, denoted by $X_{i}$ and $X_{j}$. The condition entropy of $X_{i}$ and $X_{j}$ is defined as

(2) $$H\left(X_{i}|X_{j}\right)=H\left(X_{i},X_{j}\right)-H\left(X_{j}\right),$$

which quantifies correlation between $v_{i}$ and $v_{j}$. With the decrease of conditional entropy, the correlation of images captured by $v_{i}$ and $v_{j}$ increases.

Each video sensor reports its focal length and FoV parameters to the sink. After receiving these parameters, the sink calculates $r_{i,j}$ between any two video sensors using Equation (1) and distributes corresponding results to each node. Neighbor discovery is provided by the common control channel signaling periodically. By this signaling, all nodes know one-hop and two-hop neighbors and their vacant channels. The clustering process is generated when an event is detected. Event-detecting nodes become member nodes directly. Then, a request message is sent by these member nodes to non-member one-hop neighbors. Upon receiving this request, each non-member node determines whether they join clustering. The condition for becoming member nodes depends on the correlation between nodes. The member nodes newly elected send a message to their non-member one-hop neighbors, and the process continues until the event is not within nodes’ FOVs. Assume member node $v_{i}$ sends request message to its neighbor node, i.e., $v_{j}$. The weight between $v_{i}$ and $v_{j}$, denoted by $w_{i,j}$, is calculated by

(3) $$w_{i,j}=\frac{r_{i,j}}{H\left(X_{i}|X_{j}\right)}.$$

We introduce a correlation threshold, denoted by CT, to represent the correlation degree between two nodes. If weight $w_{i,j}$ is bigger than CT, node $v_{j}$ is chosen as a member node. We define set Υ that represents member nodes within a cluster. Clusters are formed when an event is detected and maintained until the end of the event.

A cluster head is responsible for compressing observed images and then sends compressed data to the sink. Due to the existence of PUs, the channel availability of each node is different, which causes a significant influence on the data transmission of a cluster head. Thus, how to select a cluster head to transmit compressed data without affecting PUs’ activities is of significance in CVSNs. To characterize dynamics of channel availability, we model the occupation time of PUs in each data channel as an independent and exponentially distributed alternating ON/OFF random process [11,33] with birth rate *b* and death rate *a*. The ON state indicates that the channel is occupied by PUs, whereas the OFF state implies that the channel is idle. With the PU activity model, the probability of channel *c* being occupied and idle are defined as $u_{on}^{c}=\frac{b}{a+b}$ and $u_{off}^{c}=\frac{a}{a+b}$. A larger $u_{off}^{c}$ indicates that *c* is better and more suitable for data transmission. It is assumed that each node independently estimates PUs’ activities using the above ON/OFF model instead of relying on a geo-location database [34].

Eligible nodes within the scope of an event participate in DCS. For cluster head election, it is necessary to separate transmission and compression as much as possible. The goal is to balance energy consumption and reduce the probability of congestion. This corresponds to the following optimization problem:

(4) $$v_{i}^{*}=\arg\;\max_{v_{i}\in Y}E_{i},$$

subject to:

(5) $$d\left(v_{i},s\right)<d\left(e,s\right),$$

(6) $$d(v_{i},e)<r,$$

(7) $$u_{off}^{c_{i}}\geq U,$$

(8) $$\beta -\pi <\arctan\left(\frac{y_{i}-y_{0}}{x_{i}-x_{0}}\right)<\beta .$$

As shown in objective (4), the node with maximum remaining energy will be elected as a cluster head while satisfying constraints (5)–(8). Constraint (5) ensures that the distance between the cluster head and the sink is less than the distance between the sink and the event. Constraint (6) demands that the distance between the location of a cluster head and the event is less than the sensing radius of a video sensor. Constraint (7) guarantees that the probability of the channel being idle must be larger than a given channel availability threshold. Constraint (8) ensures that a chosen cluster head and the sink should be located on the same side of the event movement locus, where $\left(x_{0},y_{0}\right)$ is the location of event *e* whose moving direction is β and $\left(x_{i},y_{i}\right)$ is the coordinate of $v_{i}$. Equation (8) is composed of the inverse tangent function of $\frac{\left(y_{i}-y_{0}\right)}{\left(x_{i}-x_{0}\right)}$, which restricts the area of cluster head selection.

We provide a probabilistic guarantee of cluster head selection, in which the probability that the channel being idle should not be below γ, expressed by

(9) $$P\left(u_{off}^{c_{i}}\geq U\right)\geq \gamma ,$$

where *U* is channel availability threshold. Let $f_{off}^{c_{i}}$ be $1-u_{off}^{c_{i}}$. Equation (9) can be changed to

(10) $$P\left(f_{off}^{c_{i}}\geq 1-U\right)\leq 1-\gamma .$$

By applying the Markov’s inequality on Equation (10), we have

(11) $$P\left(f_{off}^{c_{i}}\geq 1-U\right)\leq \frac{\bar{f_{off}^{c_{i}}}}{1-U}$$

and

(12) 1−U>0.

Comparing (10) and (11), if the following inequation holds,

(13) $$\frac{\bar{f_{off}^{c_{i}}}}{1-U}\leq 1-\gamma$$

probabilistic guarantee inequation (10) for cluster head selection can be satisfied. Inequation (13) can also be expressed as

(14) $$U\leq 1-\frac{\bar{f_{off}^{c_{i}}}}{1-\gamma }$$

and, on this basis, channel availability threshold can be set to

(15) $$U=1-\frac{\bar{f_{off}^{c_{i}}}}{1-\gamma }$$

from which constraint (7) is obtained.

The probabilistic guarantee we provide can ensure the probability of the channel being idle as being more significant than a given channel availability threshold. With this method, the chosen cluster head can avoid collision with PUs’ activities as much as possible.

#### 4.1.2. Clustering Algorithm

The clustering algorithm consists of two parts, i.e., cluster head selection (shown in Algorithm 1) and cluster formation (shown in Algorithm 2). The policy in [35] is referred to improve the robustness of the algorithm in consideration of the impact of lossy links. Before the clustering algorithm is executed, each member informs its neighboring members of remaining energy and channel conditions. According to information received, the IDs of neighbouring members (including itself) that satisfy constraints (5)–(8) will be recorded in set Ψ.

In the cluster head election phase, each member can autonomously decide whether it can become a cluster head according to received messages. The head selection duration is divided into three time slots. Each member can execute the head selection rule at most twice (one time slot at a time). The operations performed in each time slot are summarized as follows:

1. In the first time slot, each member performs cluster head selection for the first time. If a member has maximum energy, it broadcasts a *DECLARE* message (see line 3) to its neighboring members and becomes a candidate cluster head. The candidate cluster head is silent until the end of the cluster head selection process. Otherwise, a member does not send any messages.
2. In the second time slot, the execution process of each member except candidate cluster heads is as follows. Upon receiving a *DECLARE* message, a member broadcasts an *ACK* message to its neighbors to inform them that it has associated with one cluster head. Variable *t* is introduced to indicate whether it is the first time a *DECLARE* message is received (see line 8). If a member receives more than one *DECLARE* message, it sends an *ACK* message once and puts all the IDs of the members that have broadcast *DECLARE* messages into a set that stores cluster head information, denoted by Φ. If a member only receives *ACK* messages but does not receive any *DECLARE* messages (there is no candidate cluster head in its neighborhood), it remains silent and removes the neighboring members that have sent *ACK* messages from set Ψ.
3. In the third time slot, those silent members in the second time slot perform head selection again.

**Algorithm 1:** Cluster Head Selection

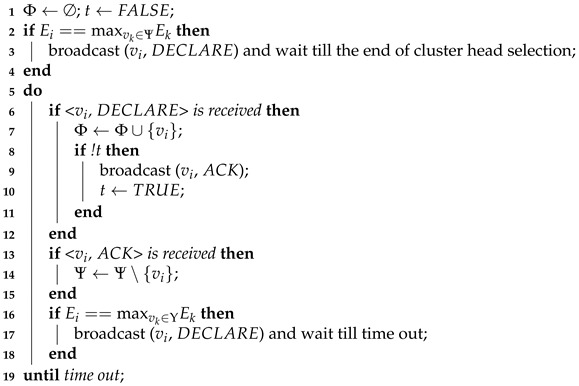



Next, we explain the cluster formation process. Those members that have been covered by multiple cluster heads follow the rule to associate themselves with a cluster head that has maximum energy. If there is a member node not covered by any cluster heads, the node declares itself as a cluster head.

**Algorithm 2:** Cluster Formation

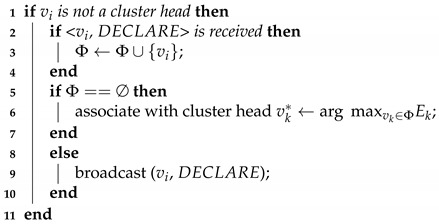



### 4.2. Collaborative Node Selection with SCS

The number of measurements *m* required in DCS is preset according to the signal sparsity which is often unknown or even time-varying. If a preset signal sparsity is higher than the actual situation, a redundant measurement sequence is generated. The redundant measurement sequence not only does not further improve CS performance but also results in additional compression measurements and transmission data redundancy. Conversely, the preset signal sparsity may be too low with less measurement sequence, making the detection accuracy requirement challenging to meet. With the support of the SCS technique [32] unaware of signal sparsity, we can determine the number of measurements required to satisfy the reconstruction error, and choose suitable nodes to form clusters to carry out DCS. By minimizing redundant information, such a technique not only saves energy but also helps provide QoS in routing.

Benefiting from SCS, we can decide whether enough samples have been obtained, with which reconstruction error can be limited to a specified threshold. Assume the sparsity of signal *x* is *k*, SCS first obtains an initial measurement vector $y^{m}={\left(y_{1},\cdots ,y_{m}\right)}^{\prime }\in R^{m}$ based on experience, and then accept *G* additional measurements. The distance between the current reconstruction $x^{m}$ of *m* measurements and the affine space $H_{m+G}$ determined by m+G measurements is

(16) $$d\left(x^{m},H_{m+G}\right)={\left(\Phi^{m+G}\right)}^{+}\left(\Phi^{m+G}x^{m}-y^{m+G}\right),$$

where $\Phi^{m+G}\in R^{(m+G)\times k}$ represents the measurement matrix, and ${\left(\Phi^{m+G}\right)}^{+}$ is a pseudo inverse matrix of $\Phi^{m+G}$. The reconstruction error of *m* measurements with probability at least $1-\frac{1}{a^{2}}$ is as follows:

(17) $$\|x-x^{m}{\|}_{2}\leq C_{G}^{a}d\left(x^{m},H_{m+G}\right).$$

In Equation (17), the value of $C_{G}^{a}$ is computed by

$$\sqrt{\frac{Q-2}{G-2}}+a\sqrt{\frac{Q-2}{G-2}-\frac{Q}{G}},$$

where Q=k−m; $C_{G}^{a}d\left(x^{m},H_{m+G}\right)$ is the estimated value of the reconstruction error. In the following, we stop taking new measurements once the estimated value of reconstruction error in (17) falls below a desired threshold. Otherwise, taking *G* as the step and increasing the number of measurements sequentially until the reconstruction error is under a specified reconstruction threshold, we get measurement vector $y^{m+sG}$, where s=0,1,…,S.

After *m* collaborative nodes are selected, a cluster head compresses their observed images jointly and sends compressed data to sink hop by hop.

### 4.3. Distributed QoS Routing

Each node distributively selects the optimal next hop with the objective of minimizing energy consumption and satisfying QoS requirements in delay and reliability.

Suppose a cluster head needs to forward a video frame to the sink. We define the set of selectable neighbors that are closer to the sink than itself and on the same side of the track of the event with the sink by $F_{i}$. The next hop node $v_{j}$ is selected from $F_{i}$ according to the following rules:

$$\mathrm{Given}:v_{i},v_{j}\in F_{i},c\in C_{i,j},r_{i,j}^{c}\in R$$

$$\mathrm{Find}:v_{j}^{*},c^{*}$$

(18) $$\mathrm{Minimize}:\;E\left(\frac{L}{p_{i,j}^{c}},d\left(v_{i},v_{j}\right)\right),$$

subject to:

(19) $$\frac{L}{p_{i,j}^{c}\cdot r_{i,j}^{c}}<T_{i,j},$$

(20) $$p_{i,j}^{c}\geq P_{i,j}.$$

The local optimal next hop $v_{j}^{*}$ is the node that results in the minimum energy consumption under local delay and local reliability constraints. A channel transmission rate, i.e., $r_{i,j}^{c}$, is chosen from a set of channel transmission rates *R* belonging to $\left\{R_{1},R_{2},...,R_{n}\right\}$.

The objective of (18) is to minimize energy consumption for transmitting a packet of *L* bits data with channel transmission rate over a distance of $d\left(v_{i},v_{j}\right)$. Constraint (19) is the local delay requirement. Constraint (20) is the local reliability requirement.

#### 4.3.1. Energy Consumption

The model in [36] is used for quantifying the energy dissipation of data communication. Suppose that one sensor node transmits *l* bits of data over a distance *d* to another node. The energy consumption for data transmission is

(21) $$E_{t}\left(l,d\right)=l\cdot E_{elec}+\varepsilon_{amp}\cdot l\cdot d^{\alpha }$$

while the energy consumption for receiving these bits is

(22) $$E_{r}\left(l,d\right)=l\cdot E_{elec}.$$

The electronics energy, denoted by $E_{elec}$, is the energy needed by the transceiver circuitry to transmit or receive one bit, whereas $\varepsilon_{amp}$ is a constant for communication energy. The total energy consumption for transmitting and receiving *l* bits over a distance *d* is given by

(23) $$E\left(l,d\right)=E_{t}\left(l,d\right)+E_{r}\left(l,d\right)=2\cdot l\cdot E_{elec}+\varepsilon_{amp}\cdot l\cdot d^{\alpha }.$$

Apart from the energy consumed by data transmission and reception, the energy consumed by in-network processing is also considered. This part of energy consumption can be modeled as a function of supply voltage. Suppose the execution of a task consisting of $N_{cyc}$ clock cycles, the energy consumption for processing is estimated as

(24) $$E_{proc}\left(N\right)=N_{cyc}C_{total}V_{dd}^{2}+V_{dd}\left(I_{0}e^{\frac{V_{dd}}{nV_{T}}}\right)\left(\frac{N_{cyc}}{f}\right).$$

The first term in (24) is the switching energy, where $C_{total}$ is the total capacitance switched by the computation per cycle, and $V_{dd}$ is the supply voltage. The second term stands for the leakage energy, where *f* is the clock speed, and $I_{0}$, *n* are processor-dependent parameters [37].

#### 4.3.2. Local Reliability Guarantee

Consider a multi-channel CVSN. The existence of PUs makes each channel have two states: busy and idle, and their transmission rates are different. A redundancy scheme is incorporated in transmission to adapt to varying wireless channel conditions. A cluster head adds an appropriate amount of redundancy to the packet according to the delivery rate of the link. Higher link delivery rate means less redundancy added to packets.

We consider the CSMA MAC protocol, with which the network is characterized by multihop wireless lossy links. To calculate reliability, we use the packet delivery ratio, the percentage of packets successfully sent to the destination. If we require that each hop on a route should provide the same level of reliability, the required packet delivery ratio from $v_{i}$ and $v_{j}$, can be estimated as

(25) Pi,j=P11N^i,sN^i,s,

where *P* is the required packet delivery ratio given by the applications and ${\hat{N}}_{is}$ can be calculated by

(26) $${\hat{N}}_{i,s}=max\left(\left\lceil \frac{d\left(v_{i},s\right)}{d_{hop}}\right\rceil ,1\right).$$

In Equation (26), the average single hop distance, denoted by $d_{hop}$ can be estimated as the arithmetic mean of the distance between $v_{i}$ and all its forwarding neighbor nodes, i.e.,

(27) $$d_{hop}=\frac{\sum_{v_{j}\in F_{i}}d\left(v_{i},v_{j}\right)}{\left|F_{i}\right|}.$$

In a layered wireless network protocol stack, video frames are split into multiple packets for transmission [38,39]. Next, we explain how to obtain the required packet delivery ratio, denoted by *P*. One important problem to be considered in the transmission process of encoded data is whether packets can be decoded successfully at the sink. Hence, the probability that a video frame is successfully decoded, denoted by PD, is introduced as a metric to evaluate reliability. We introduce a frame decodable threshold [40], denoted by DT, to represent the percentage of packets that are needed to decode a frame. Suppose that a video frame *X* is packed into *n* packets. The probability that at least DT percent of packets are successfully delivered/received, denoted by ω(X), is estimated through *n*, DT and *P*, expressed by

(28) $$\omega \left(X\right)=\omega \left(n,DT,P\right)=\sum_{i=\left\lceil n\cdot DT\right\rceil }^{n}\left(\begin{array}{c} n\\ i \end{array}\right)\cdot P^{i}\cdot {\left(1-P\right)}^{n-i}.\;$$

A video frame will be decodable if at least DT percent of the packets are received by the sink. If a video sensor has generated a video frame *X*, the probability that *X* is successfully decoded is expressed as

(29) $$P_{D}\left(X\right)=\omega \left(X\right)=\omega \left(n,DT,P\right).$$

The probability that the frame is successfully decoded $P_{D}\left(X\right)$ is correlated with the process of compressed sensing of data, which is limited by the decoded effect of packet, i.e.,

(30) $$P_{D}\left(X\right)=1-\frac{{∥x-x^{m}∥}_{2}}{{∥x∥}_{2}}.$$

In this work, given a required $P_{D}\left(X\right)$ from an application, the number of packets for X(n), DT, and *P* is estimated and assigned to each packet.

#### 4.3.3. Local Delay Guarantee

Local delay requirement also needs to be considered in data transmission. A node knows its neighbors, available channels and transmission rates when it chooses a next-hop node. Thus, it can choose one from a set of channel transmission rates $\left\{R_{1},R_{2},\ldots ,R_{n}\right\}$. The higher the channel transmission rate, the shorter the time data transmission spends.

A geographic mechanism is used to map end-to-end delay requirements to local delay requirement. Suppose a packet with a length of *L* at $v_{i}$ needs to be delivered to the sink within delay *T*. The local delay constraint, denoted by $T_{i,j}$, is expressed as

(31) $$T_{i,j}=\frac{T}{{\hat{N}}_{i,s}}.$$

The transmission delay for a packet from $v_{i}$ to $v_{j}$ is $\frac{L}{p_{i,j}^{c}\cdot r_{i,j}^{c}}$, where $\frac{L}{p_{i,j}^{c}}$ is the packet length after redundancies are added.

#### 4.3.4. Protocol Operation

The cluster-based distributed compressed sensing approach for QoS routing is described as follows. After the establishment of network topology, member nodes and the cluster head is selected according to the correlation of adjacent video nodes. Then, *m* collaborative nodes are found out from member nodes to participate in DCS. The images observed by collaborative nodes are jointly compressed at the cluster head. Lastly, the cluster head sends compressed data to the sink with the objective of minimizing energy consumption and satisfying QoS requirements in delay and reliability. Considering the problem of energy consumption and load balance in the event region, we choose nodes in the area out of the event area to transmit data.

The next-hop is selected by performing Algorithm 3. The channels of $v_{j}$ in $F_{i}$ are put in a set *J* only if the local delay and reliability requirements can be satisfied. By computing (18), the optimal channel of $v_{j}$ is selected from the channels selected above. After all nodes in $F_{i}$ find their optimal channel, the node that results in the lowest energy consumption among all nodes in $F_{i}$ is selected as a forwarder. The channel $c_{i,j*}^{*}$ of the optimal node $j^{*}$ becomes transmission channel correspondingly.

**Algorithm 3:** QoS-Guaranteed Next Hop Selection

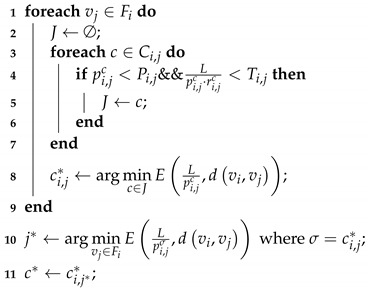



## 5. Performance Evaluation

This section involves thorough performance analyses and evaluation of the proposed CDCS in simulation methodology. We first analyze and evaluate the compression efficiency of DCS by reconstruction error rate and PSNR. We then provide reconstructed images to compare performance differences visually. Finally, we test the performance of the proposed routing algorithm that works together with CDCS. The parameter settings mainly refer to Refs. [25,41]. The default parameters are summarized in Table 1.

We evaluate the performance of CDCS under varying traffic load and QoS requirements. For performance comparison, we choose two baselines, i.e., multi-channel MMSPEED (referred to as M3SPEED) and CDCS without correlation-aware design (referred to as DCSR). M3SPEED supports service differentiation and probabilistic QoS guarantees using probabilistic multipath forwarding. Considering that it fails to support CR, we extend its capabilities concerning spectrum sensing and channel selection to make it comparable.

Three experiments are designed to study compression efficiency, energy efficiency, and QoS provisioning. All the data presented include the average of 100 random experiments to improve the accuracy of experimental results. The performance metrics to be examined are as follows:

1. Reconstruction error rate: the ratio of image reconstruction error to the original image.
2. Peak-signal-to-noise ratio (PSNR) in the unit of decibel (dB) of reconstructed images, calculated by
  $$\;10{log}_{10}\frac{255^{2}}{\frac{1-P_{D}\left(X\right)}{256\cdot 256}}.$$
3. Reconstructed images achieved by CDCS and DCSR.
4. Reliability: the proportion of packets received at the sink out of the total number of packets that satisfy different QoS requirements.
5. Energy consumption: the average energy utilization for a received frame at the sink.
6. Delay: the average end-to-end delay for delivering packets to the sink.

### 5.1. Compression Efficiency

**Figure 5:**
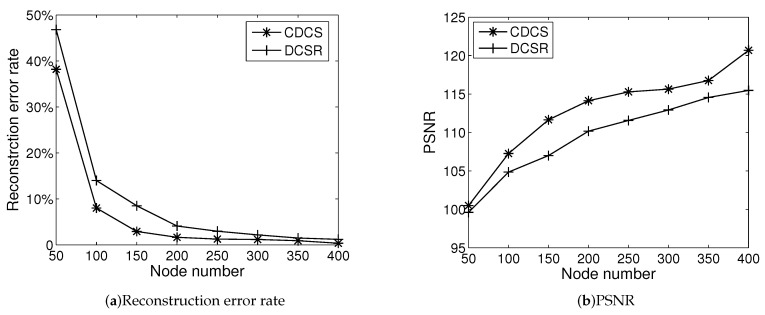
Compression efficiency.

Experiment I looks into the effect of node number on video compression efficiency. We deploy various numbers of video sensors in a field. The sink records their FoV parameters. The video sensors’ sensing radius is set to be 30 m, and the offset angle is set to be $60^{\circ }$. The locations and sensing directions of each video sensor are fixed. Each sensor captures one image at each deployment, and the size of each image is 256 × 256. Each image is segmented into blocks of 8 × 8 before performing DCS. We first encode original images by DCS. Then, we reconstruct the images received at the sink. We process the image through matrix operations in MATLAB. We obtain images’ reconstruction error rate using (30). Figure 5 shows the results of DCS compression efficiency under the different number of nodes. We give the reconstruction error rate and PSNR achieved by CDCS and DCSR.

When an image *X* has a sparse representation in one basis, it can be reconstructed by a small number of projections onto a second basis that is incoherent with the first. The reconstruction error rate of *X* is calculated by $1-P_{D}\left(X\right)$. From Figure 5a, the reconstruction error rate of image declines with the increase of node number. When the node number in the network is high, the reconstruction error tends to zero, and the image shows a good reconstruction effect. Without considering correlation, nodes are clustered randomly, and some nodes that have not observed the objects may appear in the cluster. Because there is no object in the observation of these nodes, the compression for the object cannot be realized. As a result, the number of nodes participating in DCS decreases and the reconstruction error rate increases. The increase in the number of chosen nodes increases the probability of the node to observe the object, making up for the disadvantage without consideration of the correlation to a certain extent. Meanwhile, there exists Gaussian noise while nodes acquire images. When the number of nodes increases, the intensity of the signal increases gradually, resulting in a larger PSNR.

**Figure 6:**
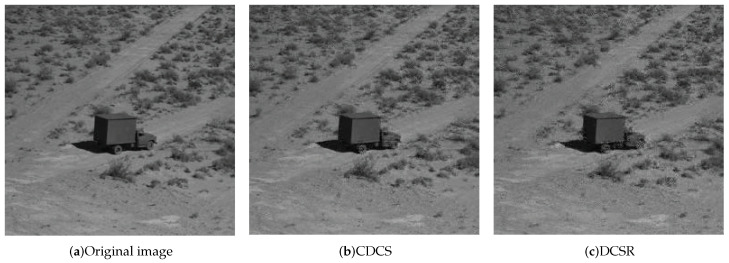
Reconstruction.

We present reconstructed images shown in Figure 6 to more intuitively compare the effects of image reconstruction. This result corresponds to the PSNR values in Figure 5b when the number of nodes is 20. We can see that CDCS achieves a better reconstruction effect than DCSR. By carefully comparing the front and rear wheels of the vehicle, it can be seen that the image distortion of the CDCS is lower than that of DCSR. One main reason is that CDCS can drive enough nodes to participate in compression.

### 5.2. Energy Efficiency

**Figure 7:**
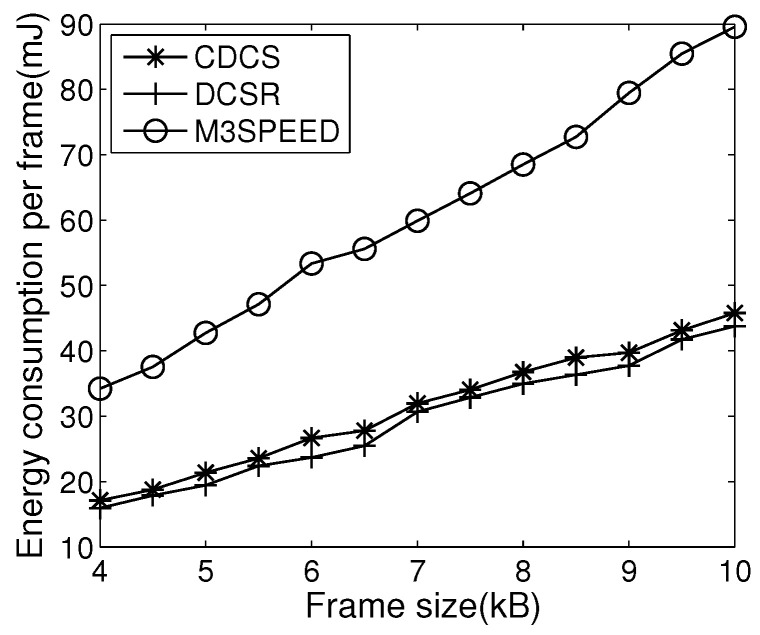
Energy consumption.

The purpose of the next experiment is the energy utilization efficiency of the proposed algorithm, where the deadline is set to be 1 s, and the required packet delivery ratio (i.e., *P*) is set to be 0.85. Figure 7 illustrates the result of energy consumption.

The primary energy consumption consists of communication energy and the energy for processing the video frames. Compared with M3SPEED, both CDCS and DCSR require energy consumption for in-network processing (i.e., encoding local video frames). However, the primary problem for M3SPEED is that it lacks control for redundant data, so there is much energy consumption in routing decisions. The proposed CDCS approach aims to reduce energy consumption by reducing the transmission of redundant information and selecting energy-efficient next hops. Hence, both CDCS and DCSR consume less energy in contrast to M3SPEED. Without consideration of FoV correlation, DCSR has less number of nodes that observe an object in a cluster. Due to limited video compression, the energy consumption of DCSR is less than CDCS.

### 5.3. QoS Provisioning

**Figure 8:**
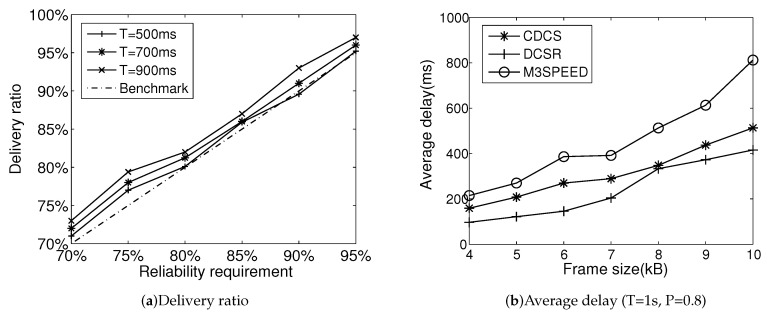
QoS provisioning.

Experiment III looks at the received video information after setting various constraints of delay and reliability. Figure 8a gives the frame delivery under reliability requirements for different deadlines. For each reported image frame, we count the number of received packets within the deadline. If the percentage of received packets for a frame is above the frame decodable threshold (DT), we show that it can successfully be decoded at the sink. Based on the number of decoded frames, we can obtain the percentage of successfully decoded video frames (frame delivery ratio). From Figure 8a, CDCS can meet reliability requirements in most cases.

Figure 8b shows the result of the average packet delay with different reliability requirements. CDCS can provide a lower delay for video transmissions than M3SPEED. Multimedia is resource-consuming applications. The multi-path transmission of M3SPEED brings extra traffic to a network, resulting in network performance degradation. CDCS utilizes a geographic mechanism to map an end-to-end requirement to a local delay requirement, which ensures its average delay is under delay constraint. The average delay of DCSR is lower than that of CDCS. The reason for this is that the former has less compressed data than the latter does.

Comparing Figure 7 and Figure 8b, DCSR has less energy consumption and average delay than CDCS, but it satisfies energy efficiency and delay requirement at the cost of sacrificing image quality. Thus, CDCS can provide a more effective way to improve the quality of visual information received.

## 6. Conclusions

A cluster-based distributed compressed sensing approach that combines spectrum-aware QoS routing is proposed in this work. The purpose is to minimize energy consumption while satisfying QoS requirements in delay and reliability. Based on the correlation degree among adjacent video sensors with the overlapped field of views, the video sensor that observes the object is selected as a member node. Then, SCS is used to determine enough number of collaborative nodes that can meet the requirements of the reconstruction error to participate in DCS. Finally, a QoS-guaranteed routing framework is presented in which each node chooses the optimal next hop that minimizes energy consumption in a distributed manner. Simulation results show that, by integrating FoVs’ correlation operations in DCS, CDCS can realize high energy-efficient delivery and reconstruction accuracy of visual information.

## Figures and Tables

**Table 1:** Parameter settings.

Parameters	Value
Number of video sensors	200
Number of channels	10
Transmission range	15 m
Offset angle	$60^{\circ }$
Sensing radius	30 m
Image size	256×256
$E_{elec}$	50 nJ/b
$\varepsilon_{amp}$	10 pJ/b/m${}^{2}$
α	2
Transmission rate	2 Mbps
CT	1
DT	0.8
$I_{0}$	1.196 mA
$N_{cyc}$(encoder)	2.3 Mcycles
$C_{total}$	0.67 nF
$N_{cyc}$(decoder)	0.14 M
